# PHF23-Related Prognostic Signature Modulates Immune Microenvironment and Promotes Tumor Malignancy in Glioma

**DOI:** 10.3390/ijms27062570

**Published:** 2026-03-11

**Authors:** Guoming Zhao, Xiaoqing Wang, Pengyu Yang, Peng Feng, Junqiang Dai, Liang Niu, Guoqiang Yuan, Yawen Pan

**Affiliations:** 1 Department of Neurosurgery, The Second Hospital & Clinical Medical School, Lanzhou University, Lanzhou 730030, China; zhaogm20@lzu.edu.cn (G.Z.); dhypy@163.com (P.Y.); sjwkfp@163.com (P.F.); daijunqiang91@sina.com (J.D.); niul20@lzu.edu.cn (L.N.); 2 Key Laboratory of Neurosurgery, The Second Hospital & Clinical Medical School, Lanzhou University, Lanzhou 730030, China; wxqlzu@163.com; 3 School of Life Sciences, Lanzhou University, Lanzhou 730030, China

**Keywords:** PHF23, glioma, prognostic signature, immune microenvironment, Entospletinib, HAVCR2 (TIM-3)

## Abstract

Gliomas exhibit considerable molecular heterogeneity and immunological complexity, emphasizing the need for effective biomarkers and therapeutic targets. In this study, the Chinese Glioma Genome Atlas (CGGA-325/693) and The Cancer Genome Atlas (TCGA-LGG/GBM) cohorts were used to explore the pathological role of PHD finger protein 23 (PHF23) in gliomas. Machine learning algorithms were performed to construct a PHF23-related prognosis signature (PHF23-RPS). Our analysis revealed significant upregulation of PHF23 in high-grade gliomas, while the PHF23-RPS exhibited strong predictive performance (AUC = 0.853). Two molecular subtypes were identified; Cluster 2 was characterized as “inflamed yet immunosuppressive”. This subtype displayed a tumor mutational burden (TMB) paradox, where elevated TMB failed to translate into survival benefits due to extensive M2 macrophage infiltration and checkpoint-mediated immune exhaustion. Pharmacogenomic screening and molecular dynamics simulations identified Entospletinib as a potential candidate targeting this immunosuppressive barrier, showing a stable binding affinity (−7.7 kcal/mol). Functional assays, including in vitro experiments and in vivo experiments via a male BALB/c nude mouse orthotopic glioma model (*n* = 6/group), confirmed that PHF23 silencing inhibited glioma malignancy. Our results identify PHF23 as a critical oncogenic driver in glioma and support the PHF23-RPS for risk stratification. Entospletinib may offer a potential immunomodulatory option for high-risk gliomas.

## 1. Introduction

As the predominant type of primary malignancy arising in the central nervous system, glioma remains a central focus of neuro-oncological research [1]. High-grade gliomas are notoriously associated with poor clinical outcomes. In particular, patients with glioblastoma multiforme (GBM) exhibit a median survival of less than 15 months [2] and a 5-year survival rate of only 5–10% [3,4]. Although the integration of isocitrate dehydrogenase (IDH) mutation status into the World Health Organization (WHO) classification has improved diagnostic precision [5], the efficacy of conventional treatments—including maximal safe resection, radiotherapy, and temozolomide chemotherapy—remains limited [6,7,8]. Rapid tumor recurrence is frequently observed, driven by inherent molecular heterogeneity and microenvironmental complexity [9,10]. Beyond traditional pathological classification, identifying novel biomarkers to predict treatment response and discovering new targets for precision therapy (especially immunotherapy) are imperative for patients with glioma [11,12].

Understanding the molecular basis of treatment resistance is essential for designing effective therapies. Recent studies have shown that the suboptimal drug response of gliomas is primarily attributed to distinct molecular alterations and sophisticated immunosuppressive mechanisms within the tumor microenvironment (TME) [2,5]. For example, the loss of phosphatase and tensin homolog (PTEN) [13], hypoxic metabolism signaling [14,15], and activation of the exosome-mediated insulin-like growth factor 2 mRNA-binding protein 3 (IGF2BP3) pathway [16] drive tumor-associated macrophages (TAMs) toward an immunosuppressive phenotype. These factors polarize TAMs into pro-tumor phenotypes, thereby hindering effective immunotherapy. This intrinsic heterogeneity limits the implementation of unified treatment regimens, underscoring the urgent need for advanced methodologies to stratify patients more effectively [14]. Unsupervised clustering through multi-omics analysis enables the distinction of immune subtypes and TAM polarization states, facilitating accurate patient stratification [17]. Integrative bioinformatics approaches can identify patients with different immune checkpoint and myeloid characteristics, which is beneficial for the development of precision neuro-oncology and prognosis prediction [18,19].

In the process of epigenetic regulation, proteins containing plant homeodomain (PHD) fingers serve as important histone modification readers that mediate chromatin remodeling and transcriptional regulation [20,21]. PHD finger protein 23 (PHF23) belongs to this prominent family of epigenetic readers. By modulating gene expression and protein stability, PHF23 has been increasingly implicated in the pathogenesis of various systemic malignant tumors [22,23]. Emerging evidence suggests that dysregulated epigenetic regulators, including PHF23, can profoundly reshape the glioma microenvironment [24]. These alterations frequently synergize to drive TAM polarization and maintain an immunosuppressive niche [25]. While immune checkpoint inhibitors have revolutionized the treatment of many solid tumors [26], their efficacy in glioma remains limited. This is largely due to the complex interaction between epigenetic regulators and checkpoint molecules, such as hepatitis A virus cellular receptor 2 (HAVCR2, also known as TIM-3) [25,27,28]. Whether PHF23 as an “epigenetic reader” to facilitate immune evasion by regulating the transcriptional program of checkpoint genes remains a critical open question. Although PHF23 plays important roles in other cancers, its expression pattern, clinical significance, and potential immunomodulatory mechanisms in glioma remain largely unexplored. Given the urgent need for reliable biomarkers to guide patient stratification, investigating whether PHF23 serves as a key oncogenic driver shaping the tumor immune environment and promoting tumor aggressiveness is of paramount importance.

In this study, we systematically analyzed the expression patterns of PHF23 across the Chinese Glioma Genome Atlas (CGGA) and The Cancer Genome Atlas (TCGA) cohorts to improve the accuracy of glioma prognosis prediction. Machine learning methods were employed to identify a PHF23-associated gene set, based on which a PHF23-related prognostic signature (PHF23-RPS) was constructed. This signature effectively stratified patients into distinct risk groups according to tumor-specific molecular characteristics. We further extensively investigated the mechanistic association of PHF23 with TAM polarization, immune checkpoint profiles, and the glioma immune microenvironment. To experimentally validate the functional role of PHF23 in glioma progression, both in vitro and orthotopic in vivo experiments were performed. The comprehensive workflow of this study design is illustrated in Supplementary Figure S1. This study aims to elucidate the clinical utility of PHF23 as a predictive biomarker and a potential therapeutic target for personalized immunotherapy in glioma patients.

## 2. Results

### 2.1. PHF23 Is Upregulated and Correlates with Clinical Progression and Poor Prognosis in Glioma

**Figure 1:**
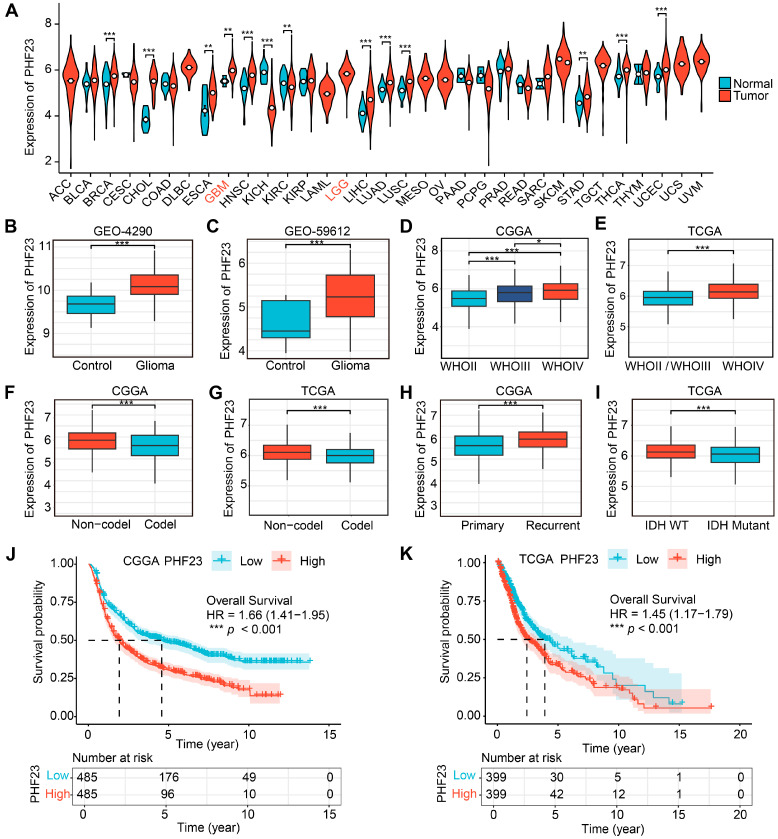
*PHF23* shows high expression and correlates with advanced tumor features and poor outcomes in glioma. (**A**) *PHF23* expression across multiple cancers and normal tissues in the The Cancer Genome Atlas (TCGA) database (Wilcoxon test). (**B**,**C**) *PHF23* expression in normal brain and glioma tissues in Gene Expression Omnibus (GEO) datasets (Wilcoxon test). (**D**,**E**) *PHF23* expression across World Health Organization (WHO) grades II–IV in the Chinese Glioma Genome Atlas (CGGA) cohort (Kruskal–Wallis test followed by pairwise Wilcoxon tests with Benjamini–Hochberg correction) and TCGA cohort (Wilcoxon test). (**F**,**G**) *PHF23* expression in 1p/19q codeleted versus non-codeleted tumors in the CGGA and TCGA cohorts (Wilcoxon test). (**H**) *PHF23* expression in primary versus recurrent gliomas in the CGGA cohort (Wilcoxon test). (**I**) *PHF23* expression in isocitrate dehydrogenase wild-type (IDH-WT) versus IDH-mutant tumors in the TCGA cohort (Wilcoxon test). (**J**,**K**) Kaplan–Meier curves of overall survival based on median *PHF23* expression in the CGGA and TCGA cohorts (Log-rank test). * *p* < 0.05, ** *p* < 0.01, *** *p* < 0.001.

We first investigated *PHF23* expression across diverse cancers within the TCGA database. As shown in Figure 1A, *PHF23* expression was significantly elevated in 11 cancers, including GBM, lung cancer (LUAD/LUSC), and gastrointestinal tumors (STAD, LIHC and ESCA), relative to matched normal samples. *PHF23* remained highly expressed in cohorts without matched normal controls, such as LGG, ACC, and LAML. Conversely, *PHF23* expression was reduced in only two kidney-related cohorts (KICH and KIRC, Supplementary Table S1 for abbreviations). These results indicate that *PHF23* is broadly upregulated across multiple cancer types, with particularly pronounced expression in glioma. To further validate *PHF23* expression specifically in glioma, we analyzed two independent Gene Expression Omnibus (GEO) datasets (GSE4290 and GSE59612). Consistently, *PHF23* expression was significantly higher in glioma tissues compared to normal brain samples in both GEO cohorts (Figure 1B,C).

For subsequent analyses, we harmonized the CGGA (CGGA-325 and CGGA-693) and TCGA (TCGA-LGG and TCGA-GBM) datasets into two consolidated cohorts. To maintain conciseness, these integrated cohorts are exclusively referred to as ‘CGGA’ and ‘TCGA’ throughout this study and in all associated figures. Potential batch effects between datasets were mitigated with the ComBat-seq algorithm, and the effectiveness of this correction was validated by principal component analysis (PCA; Supplementary Figure S2A–D). After excluding samples with incomplete survival information, a total of 970 patients from the CGGA cohort and 798 from the TCGA cohort were retained for subsequent analyses.

We then evaluated *PHF23* expression in relation to key clinical and molecular features. *PHF23* expression positively correlated with WHO grade, reaching peak levels in WHO IV (GBM) consistently in both cohorts (Figure 1D,E). It was also significantly lower in 1p/19q codeleted (Codel) tumors compared to non-codeleted (Non-codel) tumors across all cohorts (Figure 1F,G). Furthermore, *PHF23* was upregulated in recurrent tumors within the CGGA cohort (Figure 1H) and in IDH wild-type (WT) tumors within the TCGA cohort (Figure 1I), both of which are indicators of higher malignancy. Given its stong correlation with these malignant features, we assessed the prognostic value of *PHF23*. When stratified by median *PHF23* expression, patients in both the CGGA and TCGA cohorts with higher expression levels exhibited significantly shorter overall survival (OS), as indicated by Kaplan–Meier analysis (Figure 1J,K; *p* < 0.001).

### 2.2. Construction and Characterization of the PHF23-Related Prognostic Signature

**Figure 2:**
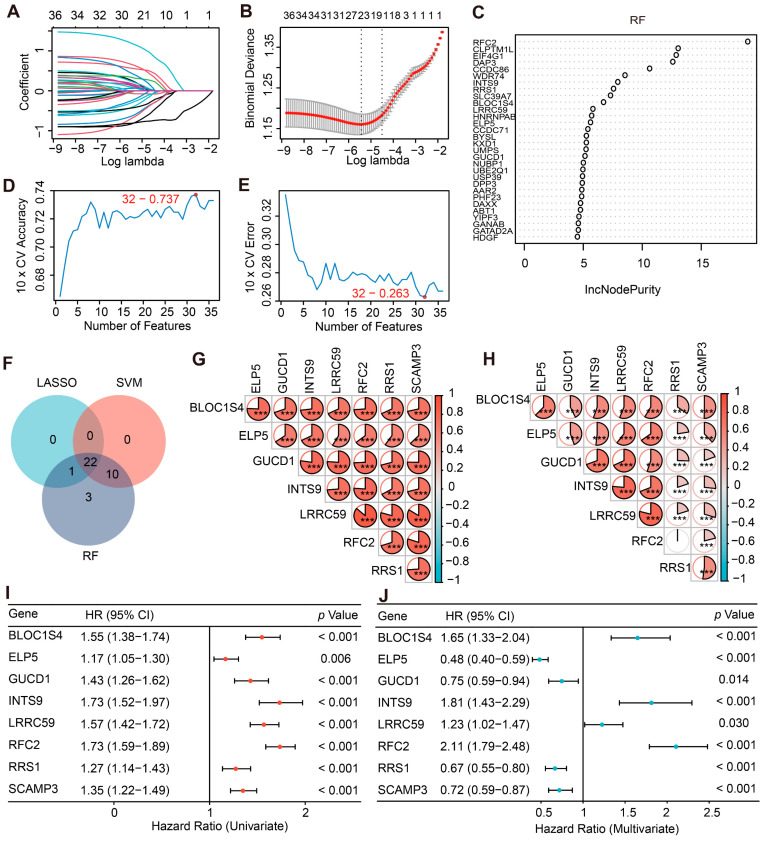
Construction of the PHF23-related prognostic signature in glioma. (**A**,**B**) Candidate genes identified by least absolute shrinkage and selection operator (LASSO) regression with 10-fold cross-validation. The colored lines represent the coefficient profiles of the genes, and the vertical dotted lines indicate the optimal values of the penalty parameter (*λ*). (**C**) Random Forest (RF) feature importance ranked by IncNodePurity. (**D**,**E**) Support vector machine-recursive feature elimination (SVM-RFE) showing accuracy (**D**) and error rate (**E**) to determine the optimal number of features. The optimal points are marked at (32, 0.737) and (32, 0.263), respectively. (**F**) Venn diagram of 22 overlapping genes from LASSO, RF, and SVM-RFE. (**G**,**H**) Correlation heatmaps of 8 hub genes in Chinese Glioma Genome Atlas (CGGA) (**G**) and The Cancer Genome Atlas (TCGA) (**H**) cohorts. (**I**,**J**) Forest plots of univariate (**I**) and multivariate (**J**) Cox regression for the 8 hub genes in CGGA. HR: hazard ratio; CI: confidence interval. *** *p* < 0.001.

*PHF23* was identified as a significant prognostic factor; however, its individual predictive performance remained limited, as indicated by areas under the curves (AUC) ranging from 0.523 to 0.643 (Supplementary Figure S2E,F). To improve its predictive ability, we constructed a multi-gene prognostic signature. Initially, 36 genes strongly associated with *PHF23* (|r| > 0.8, *p* < 0.05) were identified in the CGGA discovery cohort. Three machine learning methods were then applied to enhance feature stability and mitigate potential bias: Least Absolute Shrinkage and Selection Operator (LASSO) regression identified 23 candidate genes (Figure 2A,B); Random Forest prioritized 36 genes based on node purity (IncNodePurity > 0.5; Figure 2C); and Support Vector Machine-Recursive Feature Elimination (SVM-RFE) selected 32 genes achieving optimal accuracy (Figure 2D,E). By intersecting these results, 22 genes were consistently identified as a robust core set (Figure 2F). Subsequently, a two-stage refinement was performed to derive a parsimonious model. Multicollinearity was eliminated by iteratively removing genes with a Variance Inflation Factor (VIF) ≥ 5, resulting in 12 independent variables. These genes were then subjected to multivariate Cox regression using stepwise selection guided by Akaike Information Criterion (AIC). In the end, a final set of eight genes (BLOC1S4, ELP5, GUCD1, INTS9, LRRC59, RFC2, RRS1, SCAMP3) was selected to construct the final prognostic signature, referred to as PHF23-RPS.

Correlation analyses were conducted in the CGGA and TCGA cohorts to evaluate the relationships among genes comprising the PHF23-RPS. As shown in Figure 2G,H, the eight genes exhibited strong positive correlations (*p* < 0.001), indicating they may function synergistically to promote glioma malignancy. Subsequently, Cox proportional hazards models were employed to assess their individual prognostic value. Univariate Cox analysis indicated that nearly all constituent genes exhibited hazard ratios (HR) exceeding 1 and reached statistical significance (*p* < 0.05), confirming their association with adverse survival outcomes (Figure 2I, Supplementary Figure S3A). Interestingly, in multivariate Cox models, ELP5, GUCD1, RRS1, and SCAMP3 displayed HR < 1 in both cohorts (Figure 2J, Supplementary Figure S3B). This shift in HR values allows the PHF23-RPS to better predict glioma survival compared with individual biomarkers.

### 2.3. Validation of the Prognostic Potential of the PHF23-RPS in Glioma

**Figure 3:**
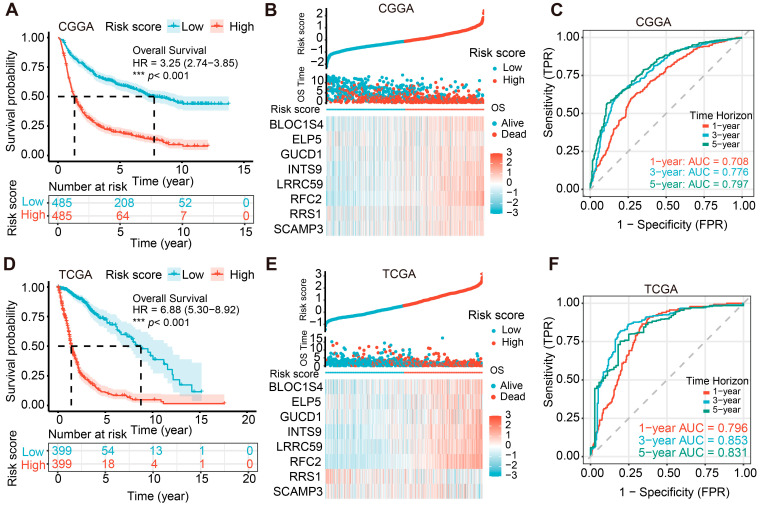
Validation of the PHF23-related prognostic signature (PHF23-RPS) prognostic performance in glioma. (**A**–**C**) Chinese Glioma Genome Atlas (CGGA) cohort: (**A**) Kaplan–Meier analysis of overall survival (OS) between high- and low-risk groups; (**B**) integrated landscape of risk score distribution, survival status, and hub gene expression; (**C**) time-dependent receiver operating characteristic (ROC) curves at 1, 3, and 5 years. (**D**–**F**) The Cancer Genome Atlas (TCGA) cohort: (**D**) Kaplan–Meier survival curves; (**E**) integrated risk score, survival status, and gene expression profiles; (**F**) time-dependent ROC curves. *** *p* < 0.001.

The PHF23-RPS risk score was calculated for all patients in the CGGA (*n* = 970) and TCGA (*n* = 798) cohorts. By employing the median risk score as the threshold, patients were stratified into high- and low-risk groups. Kaplan–Meier analysis showed that high-risk patients experienced significantly shorter overall survival compared to their low-risk counterparts in both the CGGA (HR = 3.25, *p* < 0.001; Figure 3A) and TCGA (HR = 6.88, *p* < 0.001; Figure 3D) cohorts. The risk distribution and survival status plots further demonstrated that higher risk scores strongly correlated with increased mortality rates in both the CGGA and TCGA cohorts (Figure 3B,E), supporting the signature’s robust prognostic value.

In the CGGA cohort, all eight constituent genes were significantly overexpresse in the high-risk group (Figure 3B). A similar expression pattern was observed in the TCGA cohort for seven of these genes; however, RRS1 displayed a lower expression level in high-risk patients, which is consistent with its identified protective effect in multivariate Cox analysis (Figure 3E). Moreover, time-dependent receiver operating characteristic (ROC) analyses further validated the robust performance of the PHF23-RPS in forecasting survival outcomes. In the CGGA cohort, the AUCs for 1-, 3-, and 5-year overall survival were 0.708, 0.776, and 0.797, respectively (Figure 3C), whereas in the TCGA cohort, the corresponding predictive values were even higher, reaching 0.796, 0.853, and 0.831 (Figure 3F).

### 2.4. Independent Prognostic Analysis and Clinical Application of the PHF23-RPS in Glioma

**Figure 4:**
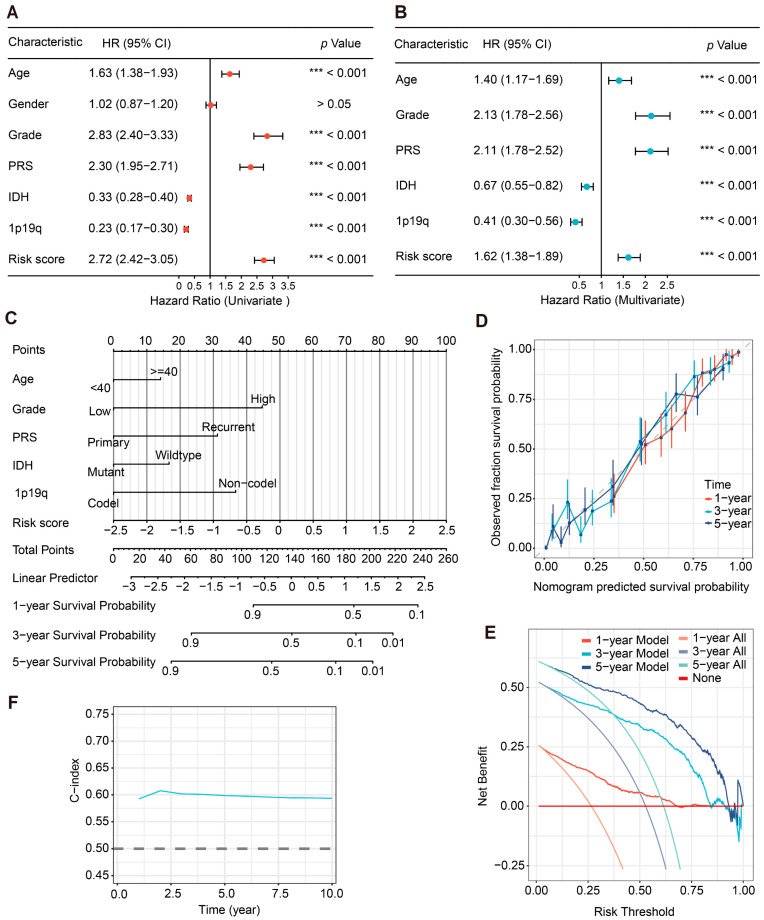
Independent prognostic analysis and clinical application of the PHF23-related prognostic signature (PHF23-RPS) in the Chinese Glioma Genome Atlas (CGGA) cohort. (**A**,**B**) Independent prognostic evaluation via univariate (**A**) and multivariate (**B**) Cox regression analyses. The *X*-axis represents the Hazard ratio. Statistical significance was determined using univariate and multivariate Cox regression analyses. (**C**) An integrated nomogram combining the risk score and independent clinical factors for survival probability prediction. (**D**) Calibration curves showing the consistency between predicted and actual survival outcomes at 1, 3, and 5 years. (**E**) Decision curve analysis (DCA) demonstrating the clinical net benefit of the integrated model. (**F**) Time-dependent concordance index (C-index) comparing the risk score and other clinical features. The dashed line at 0.5 represents the reference value for a random prediction model. *** *p* < 0.001.

To determine whether the PHF23-RPS risk score functions as an independent predictor of prognosis, both univariate and multivariate Cox proportional hazards models were applied. In the CGGA cohort, univariate analysis identified age, WHO grade, primary/recurrent status (PRS), IDH status, 1p/19q codeletion, and the risk score as significant prognostic predictors (Figure 4A). Importantly, multivariate analysis confirmed that the risk score remained an independent predictor of poor prognosis after adjusting for clinical covariates (HR = 1.62, *p* < 0.001; Figure 4B). These findings were further validated in the TCGA cohort. Age, gender, grade, IDH status, 1p/19q codeletion, and the risk score were all found to be significant in univariate analysis (Supplementary Figure S3C). More critically, the risk score independently predicted OS in multivariate analysis (HR = 1.46, *p* < 0.001; Supplementary Figure S3D), consistently demonstrating its clinical robustness across different populations.

To facilitate clinical decision-making, we developed a predictive nomogram that integrated the risk score with key independent clinical factors (age, grade, IDH, etc.) for both the CGGA (Figure 4C) and TCGA cohorts (Supplementary Figure S3E). Calibration plots demonstrated excellent agreement between the nomogram-predicted probabilities and observed survival at 1, 3, and 5 years (Figure 4D, Supplementary Figure S3F). Decision curve analysis (DCA) revealed that the integrated model achieved superior net benefit compared to individual predictors across a wide range of risk thresholds (Figure 4E, Supplementary Figure S3G). The nomogram of the TCGA cohort showed a time-dependent Concordance index (C-index) of ~0.75 over 10 years, higher than that in the CGGA discovery set (Figure 4F, Supplementary Figure S3H). These findings establish the PHF23-RPS nomogram as a robust and reliable tool for predicting long-term outcomes in glioma patients.

### 2.5. Identification and Validation of Molecular Subtypes Based on the PHF23-RPS

**Figure 5:**
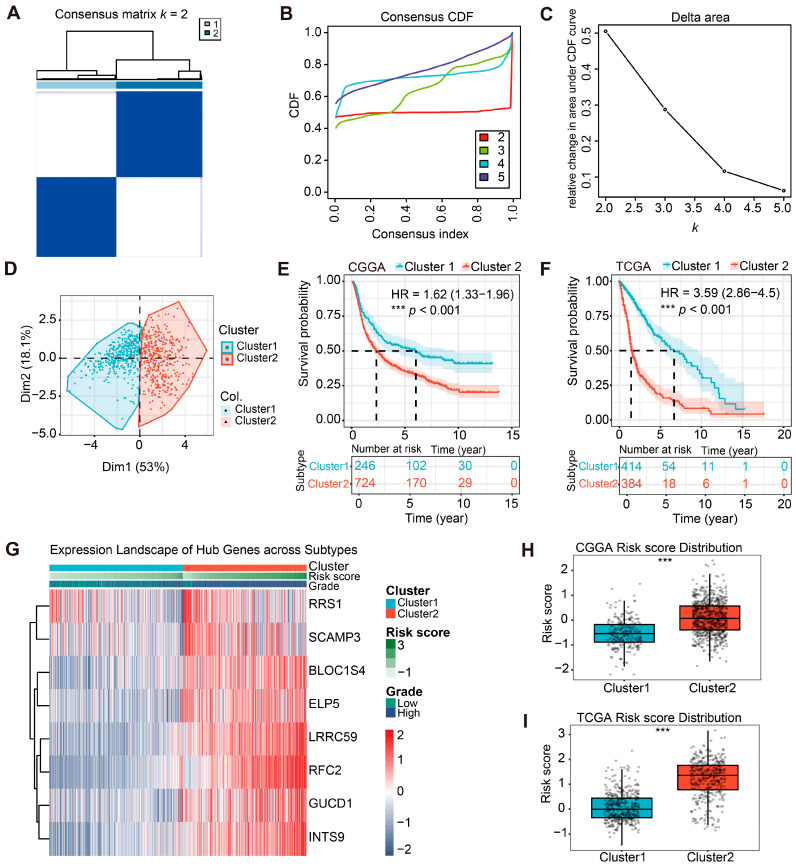
Molecular subtyping of gliomas based on the PHF23-related prognostic signature (PHF23-RPS) reveals stable clusters associated with risk and survival. (**A**–**C**) Consensus clustering analysis: (**A**) Consensus clustering matrix for the optimal *k* = 2. (**B**) Cumulative distribution function (CDF) curves for *k* = 2–5. (**C**) Relative change in the area under the CDF curve. (**D**) Principal component analysis (PCA) plot visualizing the distinct transcriptomic separation between Cluster 1 and Cluster 2. (**E**,**F**) Kaplan–Meier curves comparing the overall survival (OS) of patients in Cluster 1 and Cluster 2 in the Chinese Glioma Genome Atlas (CGGA) (**E**) and The Cancer Genome Atlas (TCGA) (**F**) cohorts. (**G**) Heatmap showing expression patterns of the 8 hub genes and their associations with cluster, risk score, and World Health Organization (WHO) grade. (**H**,**I**) Comparison of risk score distributions between Cluster 1 and Cluster 2 in the CGGA (**H**) and TCGA (**I**) cohorts (Wilcoxon test). *** *p* < 0.001.

Although the PHF23-RPS serves as a reliable continuous predictor of survival, the inherent heterogeneity of gliomas suggests the presence of distinct molecular subtypes. To elucidate these subtypes, unsupervised consensus clustering was performed on the eight signature genes using the ConsensusClusterPlus (v1.66.0) package in R software (v4.3.3). Based on the cumulative distribution function (CDF) curves and relative changes in its area for *k* = 2–5, *k* = 2 was determined as the optimal clustering number for both TCGA and CGGA cohorts (Figure 5A–C; Supplementary Figure S4A–C). Subsequent PCA revealed that Cluster 1 and Cluster 2 were clearly demarcated, indicating that the subtypes capture discernible biological differences rather than random variation (Figure 5D; Supplementary Figure S4D).

Kaplan–Meier analysis revealed that Cluster 2 exhibited significantly worse overall survival than Cluster 1 in both CGGA (HR = 1.62, *p* < 0.001; Figure 5E) and TCGA cohorts (HR = 3.59, *p* < 0.001; Figure 5F). Subtype-specific heatmaps showed that Cluster 2 predominantly composed of high-grade (WHO III/IV) gliomas and was characterized by the coordinated elevated expression of the PHF23-RPS constituent genes (Figure 5G; Supplementary Figure S4E). Conversely, Cluster 1 comprised mostly low-grade tumors with favorable survival. Taken together, these findings demonstrate that signature-driven clustering effectively captures the biological aggressiveness and clinical heterogeneity of glioma.

We found that risk scores were significantly higher in Cluster 2 than in Cluster 1 across both cohorts (*p* < 0.001; Figure 5H,I), which is consistent with their poorer survival. These findings demonstrate that the PHF23-RPS effectively reflects the major biological distinctions between subtypes. Tumors in Cluster 2 exhibit a high-risk, aggressive pheno-type, likely driven by the dysregulated PHF23-associated gene network. In comparison, Cluster 1 presents a comparatively favorable molecular profile and favorable clinical course.

### 2.6. Immune Features and Immunotherapy Prediction in PHF23-RPS-Based Glioma Subtypes

**Figure 6:**
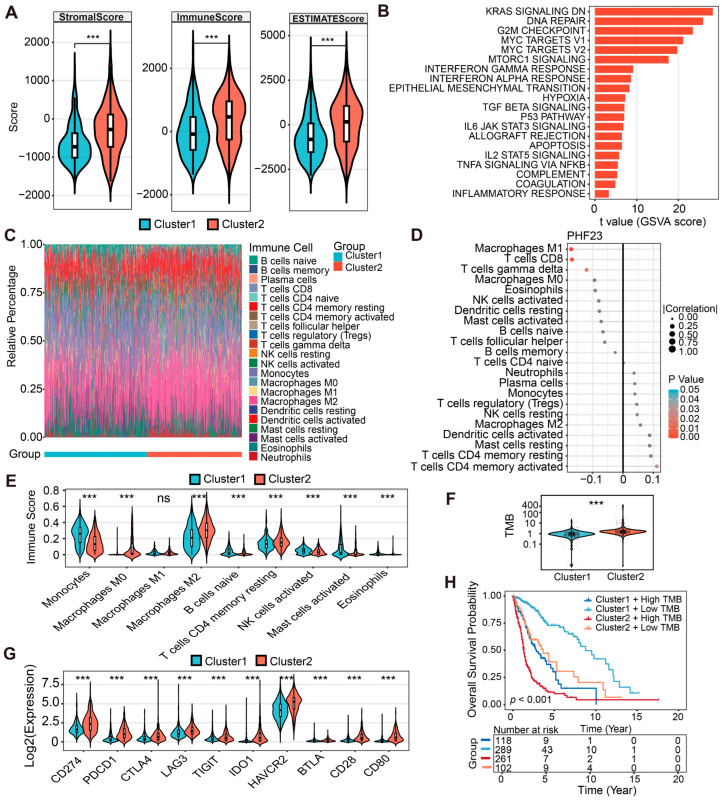
Immune landscapes and immunotherapy prediction based on PHF23-related prognostic signature (PHF23-RPS) subtypes. (**A**) Estimation of STromal and Immune cells in MAlignant Tumor tissues using Expression data (ESTIMATE) analysis comparing stromal, immune, and total scores between Cluster 1 and Cluster 2 (Wilcoxon test). (**B**) Gene set variation analysis (GSVA) enrichment heatmap showing the top 20 immune-related hallmark pathways across clusters (Wilcoxon test). (**C**–**E**) Immune cell infiltration profile: (**C**) Stacked bar plot showing the relative proportions of 22 immune cell types based on the cell-type identification by estimating relative subsets of RNA transcripts (CIBERSORT) algorithm. (**D**) Lollipop plot illustrating the correlations between PHF23 expression and various immune cell levels (Spearman correlation analysis). (**E**) Comparison of selected immune cell types between clusters (Wilcoxon test). (**F**,**G**) Immunotherapy indicators: Tumor mutational burden (TMB) (**F**) and expression of key immune checkpoints (**G**) across clusters (Wilcoxon test). (**H**) Combined survival analysis: Kaplan–Meier curves for patients stratified by both molecular clusters and TMB levels (Cluster 1/2 + High/Low TMB; Log-rank test). ns, not significant; *** *p* < 0.001.

Estimation of STromal and Immune cells in MAlignant Tumor tissues (ESTIMATE) analysis revealed that Cluster 2 exhibited significantly elevated stromal, immune, and estimates scores compared to Cluster 1, underscoring substantial differences in their TME (Figure 6A; Supplementary Figure S5A). Gene Set Variation Analysis (GSVA) of the 50 hallmark gene sets further confirmed enhanced immune infiltration in Cluster 2 (Supplementary Figure S6A,B). In both cohorts, Cluster 2 exhibited coordinated activation of major immune pathways. These included Interferon-gamma response, IL6-JAK-STAT3 signaling, TNF-alpha signaling via NF-kB, and general inflammatory response (Figure 6B). In addition to immune-related pathways, Cluster 2 also showed significant enrichment of Hypoxia, Epithelial–Mesenchymal Transition (EMT), and G2M Checkpoint pathways (Figure 6B; Supplementary Figure S6A,B). Taken together, these findings characterize Cluster 2 as an immunologically “hot” but highly malignant tumor phenotype, defined by an inflamed yet aggressive microenvironment.

We performed Cell-type Identification By Estimating Relative Subsets Of RNA Transcripts (CIBERSORT) analysis to evaluate immune infiltration. Stacked bar plots illustrated how 22 leukocyte subsets are distributed between the clusters (Figure 6C; Supplementary Figure S5C). We also found that *PHF23* expression negatively correlated with M1 macrophages and positively correlated with M2 macrophages (Figure 6D; Supplementary Figure S5D). Consistently in both cohorts, M2 macrophages were significantly enriched in Cluster 2 (Figure 6E; Supplementary Figure S5F). Correlation heatmaps revealed co-occurrence patterns among infiltrating cells (Supplementary Figure S5E). This suggests a synergistic immunosuppressive network in Cluster 2, indicating an immunosuppressive environment. Although Cluster 2 has elevated immune cell infiltration, M2 macrophages dominate its tumor microenvironment, likely promoting tumor progression and immune evasion.

In the TCGA cohort, Cluster 2 showed significantly higher tumor mutational burden (TMB) compared to Cluster 1 (*p* < 0.001; Figure 6F). This increase in TMB corresponded with a higher predicted neoantigen load, suggesting that Cluster 2 tumors have greater genomic instability. In both cohorts, cluster 2 exhibited upregulation of key immunotherapy targets, including PD-L1 (CD274), PD-1 (PDCD1), and CTLA4 (Figure 6G; Supplementary Figure S5G). Among these, HAVCR2 displayed the most pronounced differential expression, suggesting it as a potential therapeutic target for patients in Cluster 2. To further evaluate the clinical impact of these findings, we integrated molecular subtypes with TMB levels to assess clinical relevance. Kaplan–Meier analysis revealed that patients in Cluster 1 with low TMB experienced the most favorable outcomes, whereas those in Cluster 2 with high TMB suffered the most dismal survival (*p* < 0.001; Figure 6H).

### 2.7. Drug Sensitivity Prediction and Correlation with Immune Checkpoint Targets

**Figure 7:**
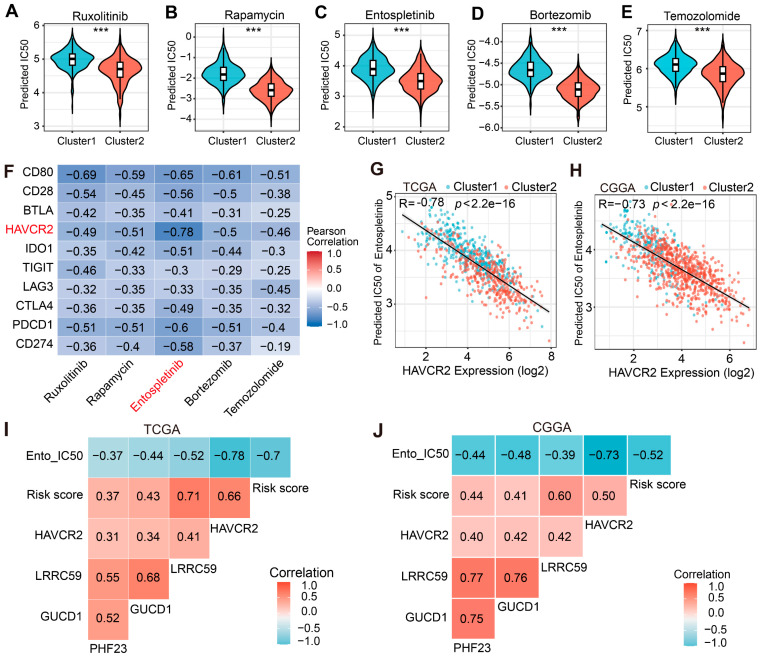
Drug screening of HAVCR2–Entospletinib. (**A**–**E**) Predicted half-maximal inhibitory concentration (IC50) values for Ruxolitinib (**A**), Rapamycin (**B**), Entospletinib (**C**), Bortezomib (**D**), and Temozolomide (**E**) across the two clusters (Wilcoxon test). (**F**) Heatmap showing correlations between key immune checkpoints and drug sensitivity (IC50). (**G**,**H**) Scatter plots of HAVCR2 expression versus Entospletinib IC50 in The Cancer Genome Atlas (TCGA) (**G**) and Chinese Glioma Genome Atlas (CGGA) (**H**). (**I**,**J**) Integrated correlation maps of PHF23, hub genes (LRRC59, GUCD1), HAVCR2, Entospletinib IC50, and the PHF23-related prognostic signature (PHF23-RPS) in TCGA (**I**) and CGGA (**J**). *** *p* < 0.001.

To identify promising therapeutic candidates, we integrated TCGA expression data with Genomics of Drug Sensitivity in Cancer (GDSC) database to predict pharmacological sensitivity across molecular subtypes. The top four immune-related drugs—Ruxolitinib, Rapamycin, Entospletinib, and Bortezomib—exhibited the strongest negative correlations with PHF23-RPS risk score. To ensure clinical relevance, we also incorporated Temozolomide (TMZ), the standard first-line chemotherapy for glioma, into our analysis. Intriguingly, the predicted half-maximal inhibitory concentration (IC50) values for all five agents were significantly lower in Cluster 2 compared to Cluster 1 within the TCGA cohort, uncovering a unique therapeutic vulnerability in this high-risk subgroup (Figure 7A–E). This pattern of enhanced therapeutic sensitivity across the five agents was also validated in the CGGA cohort (Supplementary Figure S7A–E), further supporting the potential of these agents as personalized treatment options for patients with high PHF23-RPS scores.

To identify the most clinically promising drug-target interaction, we systermatically examined correlations between immune checkpoint profiles and predicted sensitivity of these five drugs. Heatmap analysis of the TCGA cohort revealed that HAVCR2 expression exhibited the most robust negative correlation specifically with the IC50 of Entospletinib (*r* = −0.78; Figure 7F), which was also confirmed in the CGGA cohort (*r* = −0.73; Supplementary Figure S7F). Scatter plot analysis further validated this strong inverse relationship in both the TCGA (*r* = −0.78; Figure 7G) and CGGA datasets (*r* = −0.73; Figure 7H). Furthermore, integrated multi-variable correlation mapping demonstrated that the PHF23-RPS risk score was positively associated with HAVCR2 expression, while simultaneously showing a negative correlation with Entospletinib IC50 across both cohorts (Figure 7I,J). The remarkable consisitency of this negative correlation across two independent datasets strongly implies that tumors with higher HAVCR2 expression tend to exhibit enhanced predicted sensitivity to Entospletinib.

### 2.8. Molecular Docking and Dynamic Simulation Unveil the Stable Binding Mechanism of Entospletinib to HAVCR2

**Figure 8:**
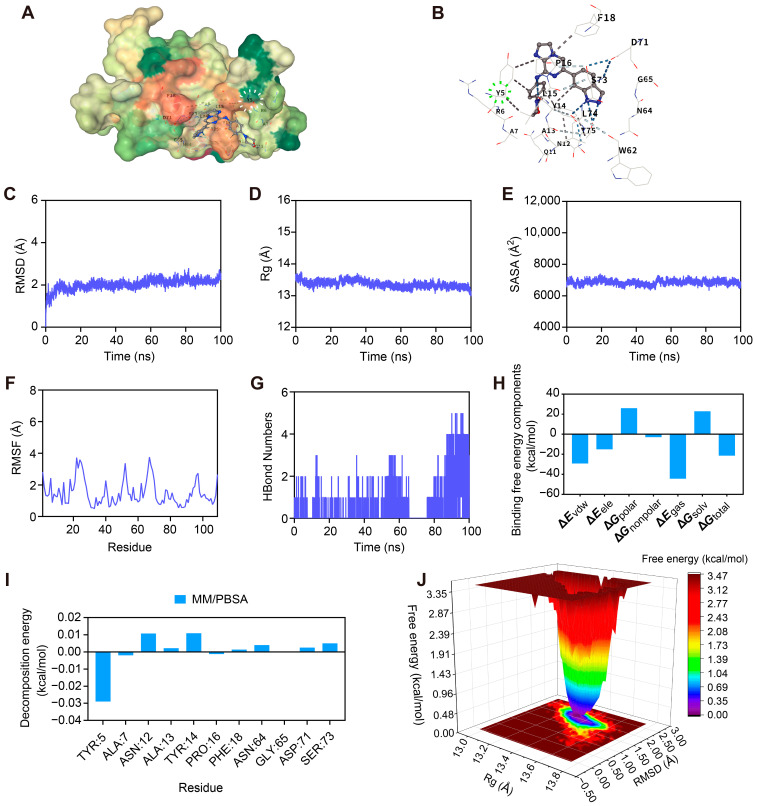
Molecular docking and molecular dynamics (MD) simulation of the HAVCR2-Entospletinib complex. (**A**) 3D surface model of Entospletinib docked in the HAVCR2 active pocket (white dashed circle highlights TYR:5). (**B**) Detailed interaction map of hydrogen bonds and hydrophobic contacts (green dashed circle marks TYR:5 interactions). (**C**–**E**) 100 ns MD simulation trajectories of root-mean-square deviation (RMSD) (**C**), radius of gyration (Rg) (**D**), and solvent-accessible surface area (SASA) (**E**). (**F**) Root-mean-square fluctuation (RMSF) of HAVCR2 residues. (**G**) Intermolecular hydrogen bond count over time. (**H**) Molecular mechanics/Poisson–Boltzmann surface area (MM/PBSA) binding free energy and thermodynamic components. (**I**) Per-residue free energy decomposition. (**J**) Free energy landscape (FEL) mapped against RMSD and Rg.

Molecular docking analysis was performed to investigate the interaction between Entospletinib and HAVCR2. The results showed that Entospletinib binds stably within the active pocket of HAVCR2 (Figure 8A). According to CB-Dock2 predictions for the optimal cavity, the ligand exhibited a strong binding affinity, characterized by a Vina score of −7.7 kcal/mol. This initial binding pose was predominantly maintained through a network of hydrogen bonds and hydrophobic interactions involving key amino acid residues within the catalytic cavity (Figure 8B).

To evaluate the structural stability of this complex over time, we performed a 100 ns molecular dynamics (MD) simulation under simulated physiological conditions. Root Mean Square Deviation (RMSD) trajectories revealed that the system reached quilibrium rapidly within the first 10 ns, maintaining a stable plateau at approximately 2.5 Å (Figure 8C). In parallel, both the radius of gyration (Rg) and solvent-accessible surface area (SASA) profiles remained steady throughout the simulation trajectory (Figure 8D,E). These findings indicate that HAVCR2 preserved its structural compactness and global integrity with no detectable unfolding events triggered by ligand binding. Residue flexibility, measured by root-mean-square fluctuation (RMSF), was generally low (<4 Å), reinforcing the overall rigidity of the complex (Figure 8F). During the simulation, we also observed consistent intermolecular hydrogen bonding—typically 1 to 2 bonds—suggesting a continuous and dynamic interaction between the ligand and the target (Figure 8G).

We subsequently employed the Molecular mechanics/Poisson–Boltzmann surface area (MM/PBSA) method to quantify binding thermodynamics. The HAVCR2-Entospletinib complex exhibited a total binding free energy of −21.44 kcal/mol, confirming a high binding affinity (Figure 8H). Residue decomposition identified specific amino acids, particularly TYR:5 and ALA:7, as the primary energetic contributors (Figure 8I). Structural mapping visually corroborates these findings; as highlighted in the 3D surface model (Figure 8A, white dashed circle) and the detailed interaction map (Figure 8B, green dashed circle), TYR:5 serves as a crucial structural anchor by forming robust intermolecular bonds with Entospletinib within the active site. Finally, mapping the free energy landscape (FEL) against RMSD and Rg revealed a distinct and concentrated energy minimum (RMSD ≈ 2.2 Å, Rg ≈ 13.3 Å), indicating that the complex successfully converges into a stable conformation (Figure 8J). Taken together, these thermodynamic and structural analyses confirm the highly stable interaction between Entospletinib and HAVCR2.

### 2.9. Knockdown of PHF23 Inhibits Glioma Cell Proliferation

**Figure 9:**
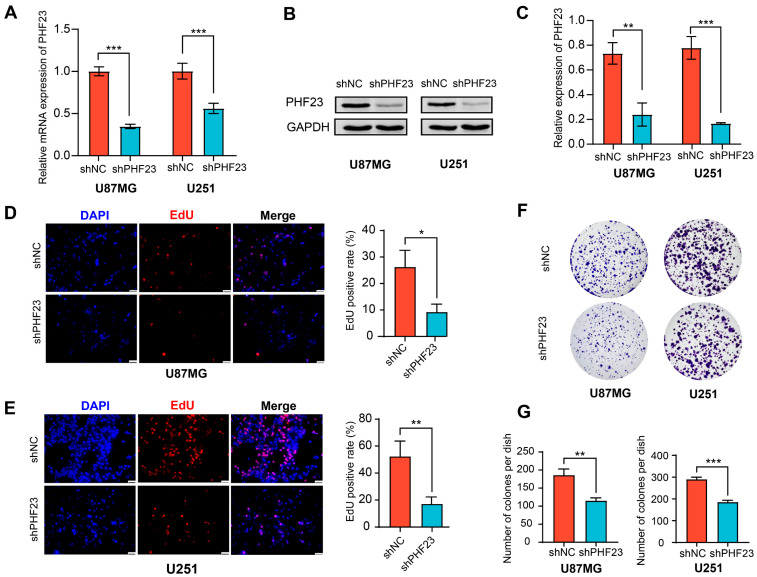
PHF23 knockdown inhibits glioma cell proliferation. (**A**–**C**) Knockdown validation: (**A**) qRT-PCR and (**B**) Western blot confirming PHF23 silencing in U87MG and U251 cells; (**C**) Quantification of protein levels normalized to GAPDH. (**D**,**E**) 5-ethynyl-2′-deoxyuridine (EdU) assay: Representative images and quantification of EdU-positive cells in U87MG (**D**) and U251 (**E**) after PHF23 knockdown versus shNC. Scale bar: 50 μm. (**F**,**G**) Colony formation assay: (**F**) Representative colonies of U87MG and U251 cells post-PHF23 knockdown; (**G**) Quantitative analysis of colony numbers. * *p* < 0.05, ** *p* < 0.01, *** *p* < 0.001.

To explore the biological function of PHF23 in glioma, loss-of-function experiments were conducted in U87MG and U251 cell lines. Knockdown efficiency of PHF23-targeting shRNA (shPHF23) was confirmed at both mRNA and protein levels. Quantitative real-time PCR (qRT-PCR) and Western blot analyses demonstrated a substantial reduction in PHF23 expression in shPHF23-transfected cells compared to scrambled negative controls (shNC) across both cell lines (Figure 9A–C). These knockdown models were used for all subsequent functional assays.

We assessed glioma cell proliferation to investigate PHF23′s potential oncogenic role. 5-ethynyl-2′-deoxyuridine (EdU) incorporation assays showed that PHF23 knockdown significantly impeded DNA synthesis, evidenced by a marked reduction in the percentage of EdU-positive nuclei in both U87MG and U251 cells (Figure 9D,E). Colony formation assays further revealed that silencing PHF23 led to reduced colony number and size relative to control cells under long-term culture conditions (Figure 9F,G). These findings indicate that PHF23 plays an important role in glioma cell proliferation and self-renewal.

### 2.10. Silencing of PHF23 Induces Apoptosis and Suppresses Migration and Invasion

**Figure 10:**
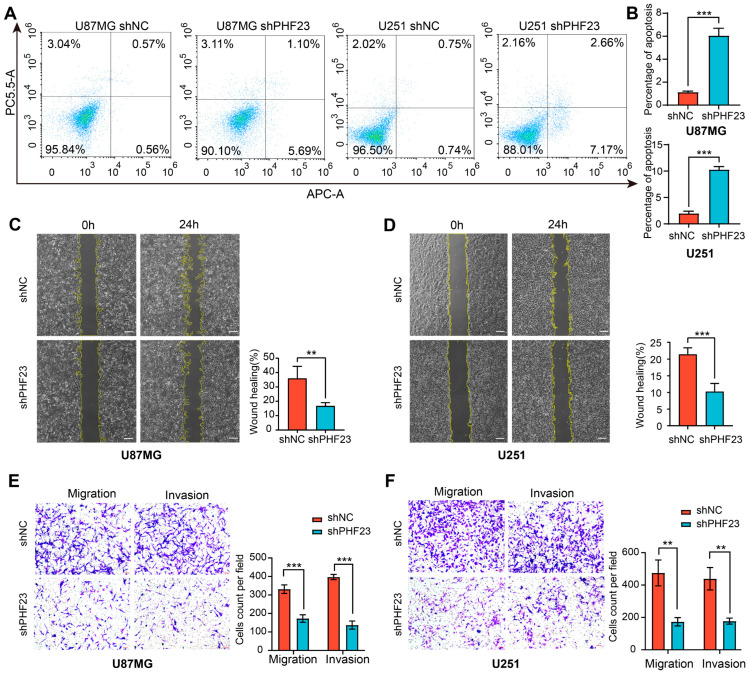
PHF23 knockdown induces apoptosis and inhibits glioma cell motility. (**A**,**B**) Apoptosis detection: (**A**,**B**) Apoptosis analysis: (**A**) Flow cytometry using Annexin V-APC/7-AAD in shPHF23 and shNC cells. The pseudo-color in the scatter plots represents local cell density, ranging from blue (low density) to red (high density). (**B**) Quantification of total apoptosis. (**C**,**D**) Wound healing assay in U87MG (**C**) and U251 (**D**) cells at 0 and 24 h. Scale bar: 50 μm. (**E**,**F**) Transwell migration and invasion assays in U87MG (**E**) and U251 (**F**) cells. Scale bar: 50 μm. ** *p* < 0.01, *** *p* < 0.001.

To further investigate the mechanism underlying the growth inhibition, flow cytometric analysis with Annexin V-APC/7-AAD staining was performed. The results revealed a clear increase in apoptotic cell populations following PHF23 silencing. In both U87MG and U251 cells, PHF23 knockdown led to a significant elevation in total apoptosis, including both early and late apoptotic fractions, compared to shNC control cells (*p* < 0.001; Figure 10A,B). This pro-apoptotic effect was predominantly driven by a higher proportion of early apoptotic cells, complemented by a modest increase in the late apoptotic subpopulation. These results suggest that PHF23 acts as a key survival factor that protects glioma cells from undergoing programmed cell death.

Wound healing assays were performed to evaluate the role of PHF23 in glioma cell motility. Cells with PHF23 knockdown exhibited significantly slower scratch closure at 24 h compared with controls, indicating reduced horizontal migration (Figure 10C,D). Transwell assays also confirmed this effect: both migration and invasion were markedly decreased in shPHF23 cells, with fewer cells traversing the membrane with or without matrigel (Figure 10E,F). These results were consistent across U87MG and U251 cell lines, demonstrating that PHF23 depletion severely impairs glioma cell motility and invasiveness.

### 2.11. Validation of PHF23 Expression in Clinical Samples and Oncogenic Role In Vivo

**Figure 11:**
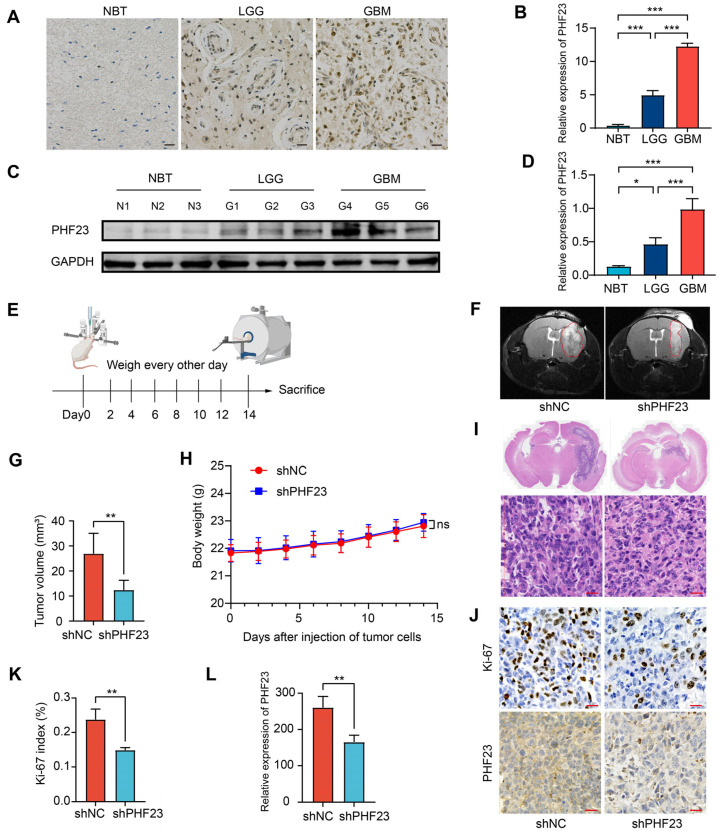
Validation of PHF23 expression in human clinical samples and its oncogenic role in an orthotopic xenograft mouse model. (**A**,**B**) Immunohistochemistry (IHC) analysis showing representative images (**A**) and statistical quantification (**B**) of PHF23 in normal brain tissue (NBT), lower-grade glioma (LGG), and glioblastoma (GBM). Scale bar: 20 μm. (**C**,**D**) Western blot analysis (**C**) and statistical quantification (**D**) of PHF23 protein levels in clinical samples. (**E**) Workflow of the orthotopic xenograft model. (**F**–**H**) Monitoring of intracranial tumor growth: (**F**) Magnetic resonance imaging (MRI) scans at day 14, (**G**) tumor volumes, and (**H**) body weight changes in shNC and shPHF23 groups. The red outlines in panel (F) delineate the boundaries of the intracranial tumors. (**I**) Hematoxylin and eosin (H&E) staining of xenograft sections showing global views and magnified morphology. Scale bar: 20 μm. (**J**–**L**) IHC evaluation of xenografts: (**J**) Representative images and (**K**,**L**) quantitative analysis of Ki-67 and PHF23 expression. Scale bar: 20 μm. Data are presented as mean ± standard deviation (SD). Statistical significance was determined by one-way analysis of variance (ANOVA) followed by Tukey’s multiple comparisons post hoc test (**B**,**D**), unpaired Student’s *t*-test (**G**,**K**,**L**); two-way ANOVA (**H**). * *p* < 0.05, ** *p* < 0.01, *** *p* < 0.001.

To validate the clinical relevance of our findings, immunohistochemistry (IHC) analysis was conducted to examine PHF23 expression in patient samples, including normal brain tissue (NBT), lower-grade glioma (LGG), and GBM. PHF23 expression was low in NBT, increased in LGG, and highest in GBM (Figure 11A,B). Consistent with these histological observations, Western blot analysis of three independent samples per group also confirmed a stepwise grade-dependent increase in PHF23 protein levels from NBT to LGG and GBM (Figure 11C,D). These results collectively demonstrate that PHF23 expression positively correlates with glioma malignancy and histological progression.

An orthotopic xenograft model was established by injecting U251-shNC or U251-shPHF23 cells into the mouse striatum to assess the role of PHF23 in tumor progression in vivo (Figure 11E). Magnetic resonance imaging (MRI) performed 14 days after implantation showed that tumors in the shPHF23 group were smaller than those in the shNC group (Figure 11F,G). Body weight remained similar between groups over the 2-week period (Figure 11H; detailed dynamic weight data are provided in Supplementary Table S2), suggesting that PHF23 knockdown reduced tumor growth without affecting overall health.

Histopathological analysis was performed after sacrifice. Whole-brain H&E staining indicated that shNC tumors were larger and infiltrative, whereas shPHF23 tumors were reduced in size (Figure 11I, upper panels). shNC tumors had increased cell density and distinct aggressive features relative to shPHF23 tumors (Figure 11I, lower panels). IHC analysis of the xenografts confirmed effective PHF23 knockdown in vivo. Ki-67, a marker of cell proliferation, showed significantly lower expression in the shPHF23 group compared with shNC (Figure 11J–L). These findings show that PHF23 knockdown led to reduced proliferation of glioma cells in the brain in vivo.

### 2.12. Schematic Illustration of PHF23-Mediated Glioma Progression and Therapeutic Potential

**Figure 12:**
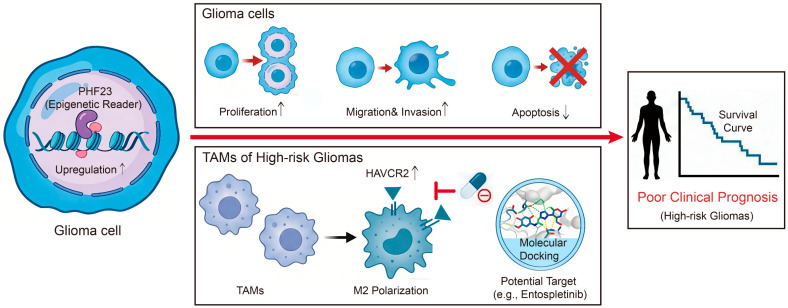
Mechanistic diagram of PHF23-mediated glioma progression and therapeutic targeting. Aberrant upregulation of the epigenetic reader PHF23 initiates a bifurcated oncogenic process. (**Left**): Overexpression of PHF23 in glioma cells serves as the upstream trigger. (**Middle**): The mechanism involves an intrinsic pathway (**top**) promoting aggressive cellular phenotypes and an extrinsic pathway (**bottom**) fostering an immunosuppressive tumor microenvironment (TME) through M2 macrophage polarization and HAVCR2 upregulation. The structural inset displays the stable molecular docking of Entospletinib into the HAVCR2 binding pocket. (**Right**): The cumulative effect of these alterations correlates with poor survival outcomes in high-risk patients. The upward arrows (↑) and downward arrow (↓) indicate increased and decreased biological processes or expression levels, respectively. The red T-bar indicates pharmacological inhibition.

To provide a comprehensive overview, we integrated the PHF23-associated regulatory network into a hierarchical mechanistic framework (Figure 12). Within this hierarchy, the aberrant upregulation of PHF23 serves as a central upstream trigger that drives oncogenesis through two distinct axes: intrinsically, it promotes malignant cell growth, invasion, and anti-apoptotic signaling; extrinsically, it remodels the TME by fostering M2-polarized macrophage enrichment. Specifically, the PHF23-RPS high-risk subgroup correlates with elevated HAVCR2 expression, positioning the PHF23 as a critical mediator of tumor immune evasion.

To identify potential therapeutic candidates, we employed artificial intelligence–assisted molecular docking followed by MD simulations. The results demonstrated that the small molecule Entospletinib forms a stable binding complex within the HAVCR2 protein pocket, supported by sustained intermolecular interactions and favorable binding affinity throughout the simulation. Collectively, the evidence reveals that PHF23 is associated with malignant progression and immune landscape remodeling, suggesting its applicability as a prognostic tool and therapeutic target in high-risk glioma.

## 3. Discussion

The prognosis for glioma patients remains dismal, primarily because molecular heterogeneity and immunosuppressive microenvironments limit the efficacy of current treatments. Consequently, identifying precise biomarkers for patient stratification has become a pressing necessity [29]. In this research, we explored the oncogenic role of PHF23 and constructed the PHF23-RPS by integrating machine learning algorithms with rigorous statistical filtering. This approach represents an effective strategy to uncover the complex molecular and immune landcapes in glioma [30,31,32]. Previous studies have shown that PHF23 drives oncogenesis in hematological malignancies [23] and lung cancers via ACTN4/ERK signaling [22]. In this study, we observed a consistent role for PHF23 in glioma, highlighting its involvement in tumor progression within the central nervous system. The functional similarity of PHF23 across multiple cancer types suggests a conserved role in promoting tumor malignancy [33]. Importantly, the PHF23-RPS reveals a strong association between PHF23-driven epigenetic regulation and the immune profile of gliomas.

The PHF23-RPS was constructed to improve the predictive performance of PHF23 as a single biomarker [34]. While PHF23 alone exhibited limited predictive accuracy, reflected by AUCs between 0.523 and 0.643, the PHF23-RPS showed significantly enhanced accuracy, with an AUC of 0.853. To ensure model stability, LASSO, random forest, and SVM-RFE were applied. Subsequent VIF screening and AIC-driven Cox regression yielded a compact 8-gene signature. This strategy reduced algorithmic bias and improved the reliability of feature selection in high-dimensional genomic data [35,36]. Interestingly, ELP5 and GUCD1 shifted from risk-associated to protective variables in multivariate analysis. This context-dependent transition suggests that the signature accounts for interactions among genes, thereby improving prognostic accuracy compared with single-gene models [37].

Multivariate analysis demonstrated that the PHF23-RPS risk score remained independently associated with prognosis, even after adjusting for clinical and molecular variables such as age, grade, PRS, IDH, and 1p/19q status. Although the 2021 WHO classification integrates IDH and 1p/19q status into glioma diagnosis [38], substantial survival heterogeneity persists among molecular subgroups [39,40]. The PHF23-RPS has the potential to refine risk stratification beyond current classification schemes. Kaplan–Meier curves confirmed that this signature effectively stratified survival outcomes among glioma patients. For clinical application, we established a nomogram that integrates the PHF23-RPS risk score for prognostic assessment [41]. Calibration analysis demonstrated excellent agreement between predicted and observed survival, with the 10-year C-index reaching approximately 0.75 in the TCGA cohort. Furthermore, DCA results suggest that the PHF23-RPS nomogram not only provides reliable prognostic predictions but may also guide personalized therapy based on individual patient risk. These findings imply that PHF23-RPS could serve as a valuable adjunct to current prognostic markers and treatment guidelines [42,43]. In multivariate analysis, the PHF23-RPS remained significant, but its effect was weaker than traditional clinical indicators, especially WHO grade. This highlights a valid concern about the clinical cost-effectiveness of routine PHF23-RPS testing. However, while relying on RNA-based panels inevitably increases costs, our signature yields critical biological insights that traditional histology fails to provide [44]. Specifically, whereas WHO grade offers limited guidance for systemic interventions, the PHF23-RPS effectively maps out the tumor’s immune landscape [45]. With the current diagnostic shift toward integrating molecular and histological data [38], the upfront costs of these RNA-based evaluations are well justified by their capacity to guide precision immunotherapies [46].

Unsupervised consensus clustering identified two stable molecular subtypes across the TCGA and CGGA cohorts [47]. Cluster 2 exhibited an ‘inflamed yet immunosuppressive’ phenotype [45]. This phenotype associates high immune activity with poorer patient survival. This may help explain the reduced survival observed in patients classified within this high-risk cluster. Cluster 2 showed significantly higher ESTIMATE scores than Cluster 1, indicating increased infiltration of immune and stromal cells within the TME. GSVA further associated this subtype with hyperactivation of IL6–JAK–STAT3 and TNF-α–NF-κB signaling pathways [48,49,50]. However, strong inflammatory signaling in glioma often reflects a pro-tumorigenic mesenchymal shift rather than effective anti-tumor immunity [51,52]. TAMs are preferentially recruited and polarized toward the immunosuppressive M2 phenotype over the cytotoxic M1 type [53]. Our correlation analysis further supports this: PHF23 expression positively correlates with M2 infiltration and inversely with M1 macrophages, suggesting that PHF23 may actively contribute to the induction of immunosuppressive polarization within the TME.

This study reveals a “TMB paradox” in glioma, where elevated mutational loads correlate with poorer survival instead of the expected immune benefit. This high TMB in Cluster 2 is likely driven by genomic instability, specifically mediated through aberrant DNA repair and dysregulated G2/M checkpoint signaling (Figure 6B). When patients were stratified by both Clusters and TMB status, the (Cluster 2 + High TMB) subgroup suffered the poorest survival. This observation aligns with the findings of Liu et al. [54], who demonstrated that elevated TMB in glioma serves as an indicator of advanced malignancy and leads to worse clinical outcomes. We propose that any potential neoantigen-driven antitumor immunity in Cluster 2 is effectively abrogated by a formidable immunosuppressive barrier. This barrier is sustained by a sharp increase in HAVCR2 expression, extensive M2 macrophage infiltration, and T cell exhaustion [55,56]. Intriguingly, HAVCR2 appears to play a more dominant role than PD-1 as a “gatekeeper” of immune suppression in glioma [57,58]. Such integrated stratification highlights the PHF23-RPS as a robust tool for capturing heterogeneity in the immune microenvironment and predicting immunotherapy response [59].

Our molecular stratification also provides actionable guidance for personalized therapy in high-risk glioma patients resistant to standard treatments. To identify the most promising candidate drugs for these patients, we integrated transcriptomic profiles with GDSC drug-response data. Our analysis revealed that Cluster 2 patients exhibited increased sensitivity to Ruxolitinib, Rapamycin, Entospletinib, Bortezomib, and TMZ, highlighting these agents as potential drug repositioning candidates [60]. While Cluster 2 patients showed sensitivity to TMZ, Entospletinib exhibited a more specific and consistent inverse correlation with the immunosuppressive marker HAVCR2 (r = −0.78 and −0.73). This suggests that Entospletinib could provide a complementary immunomodulatory benefit by addressing immune evasion barriers that often limit the efficacy of TMZ [61]. Molecular docking analysis initially indicated stable binding of Entospletinib within the active pocket of HAVCR2, with a favorable binding affinity of −7.7 kcal/mol. A subsequent 100 ns MD simulation further demonstrated the enduring dynamic stability of this complex. Based on the MM/PBSA energy decomposition, TYR:5 emerged as the predominant energetic driver for the interaction [62]. Although polar residues (such as ASN:12, ASP:71) contribute to hydrogen bonding, their slight positive free energy values merely reflect the natural desolvation penalties incurred when displacing water molecules from the binding interface [63,64,65]. Consequently, TYR:5 functions as the primary hydrophobic and electrostatic anchor, effectively sequestering Entospletinib within the active site.

Beyond this direct structural antagonism, Entospletinib is primarily recognized as a selective SYK inhibitor in hematologic cancers, such as chronic lymphocytic leukemia [66]. Interestingly, Moncayo et al. reported that SYK inhibition impairs both the proliferation and migration of glioma cells, underscoring its potential functional role in glioma [67]. In addition, SYK signaling has been reported to promote M2 macrophage polarization and tumor immunosuppression [68]. Inhibition of this pathway could promote TAMs toward an M1 phenotype [69], thereby enhancing their cytotoxic activity. Consequently, we propose that Entospletinib may exert a dual immunomodulatory effect: first, by promoting M1 polarization via SYK inhibition, and second, by alleviating HAVCR2-mediated immune evasion within the TME. These findings support a putative Entospletinib–HAVCR2 axis and highlight the therapeutic potential of Entospletinib for the treatment of high-risk gliomas.

To validate our findings, we integrated clinical sample analysis with a series of both in vitro and in vivo functional assays. Clinical sample analysis demonstrated a positive correlation between PHF23 expression and glioma grade, with PHF23 protein levels increasing progressively from NBT to LGG to GBM. Loss-of-function experiments further confirmed this finding: silencing PHF23 can inhibit DNA synthesis, colony formation, and invasive motility in glioma cell lines (U87MG and U251), while simultaneously inducing apoptosis. The key role of PHF23 in promoting tumor progression was validated in orthotopic xenograft models. Knockdown of PHF23 suppressed intracranial tumor growth and decreased the Ki-67 proliferation index, with no detectable systemic toxicity observed. The results from clinical, cellular and animal models show that PHF23 is critical for glioma proliferation and invasion. Multi-level verification lays the foundation for the oncogenic role of PHF23 and makes it a promising candidate for future epigenetic therapeutic strategies [70].

Several limitations of this study remain. First, although we confirmed the oncogenic role of PHF23 in vitro, the precise downstream signaling pathways are not yet fully understood. Second, while our multi-cohort analysis yielded consistent results, the clinical samples used for Western blot and IHC validation were limited in number. Additionally, to minimize anesthesia-related risks, tumor volume was evaluated only at the endpoint. Finally, our use of immunodeficient xenograft models constrains direct evaluation of Entospletinib’s effects on the microenvironment in vivo. Despite these limitations, our findings highlight the PHF23-RPS as a practical stratification tool to identify high-risk, immunosuppressed glioma patients who may benefit from targeted interventions. To further validate our findings, we plan to evaluate the therapeutic efficacy of Entospletinib in immunocompetent syngeneic mouse models. Such models are critical for assessing the drug’s ability to reprogram TAM phenotypes and inhibit HAVCR2-mediated immune evasion. In parallel, multi-omics approaches such as Chromatin Immunoprecipitation sequencing (ChIP-seq) and Assay for Transposase-Accessible Chromatin using sequencing (ATAC-seq) will be employed to map the comprehensive epigenetic networks regulated by PHF23. In the long term, rigorous in vivo validation and prospective clinical studies are required to determine the translational potential of the HAVCR2-Entospletinib axis in the management of high-risk gliomas.

## 4. Materials and Methods

### 4.1. Data Acquisition and Preprocessing

Transcriptomic data together with matched clinical information were collected from the Chinese Glioma Genome Atlas (CGGA; http://www.cgga.org.cn/; accessed on 8 March 2026). The CGGA-325 and CGGA-693 cohorts were combined and used as the discovery set for subsequent analyses [71,72,73]. For validation, RNA sequencing data were obtained from The Cancer Genome Atlas (TCGA; https://portal.gdc.cancer.gov/; accessed on 8 March 2026), and samples from the TCGA-LGG and TCGA-GBM projects were merged to construct a pan-glioma cohort [74]. In addition, two independent GEO datasets, GSE4290 [75] and GSE59612 [76], were downloaded from the Gene Expression Omnibus database (https://www.ncbi.nlm.nih.gov/geo/; accessed on 8 March 2026). Given the heterogeneity of platforms and sequencing batches, batch effects were corrected using the ComBat-seq algorithm implemented in the sva (v3.50.0) R package. PCA was then applied to evaluate the effectiveness of batch correction, and the distribution of risk-related features was visualized using ggplot2 (v3.5.2) in R (v4.3.3).

### 4.2. PHF23 Expression and Clinical Correlation Analysis

The pan-cancer expression landscape of *PHF23* was systematically evaluated across TCGA cohorts, followed by validation in the GEO datasets. To ascertain the clinical relevance of PHF23, subgroups were defined based on IDH mutation, WHO grade, and 1p/19q co-deletion status. Differences in expression were subsequently compared via Kruskal–Wallis or Wilcoxon rank-sum tests, depending on the group number. Finally, patients were stratified into high- and low-expression groups based on the median level of PHF23 to estimate OS via Kaplan–Meier plots and the log-rank test. All statistical analyses and visualizations were implemented using the survival (v3.8-3), survminer (v0.5.0), and ggplot2 (v3.5.2) packages, with *p* < 0.05 being considered statistically significant.

### 4.3. Identification of PHF23-Related Hub Genes via Machine Learning

To identify genes most strongly associated with *PHF23* expression, Spearman correlation analysis was performed in the CGGA discovery cohort using the psych R package. The Benjamini–Hochberg method was employed to adjust for multiple tests and limit the false discovery rate. Genes with strong co-expression with *PHF23* (absolute correlation > 0.8 and adjusted *p* < 0.05) were selected as candidate genes for subsequent analyses.

A three-step machine learning strategy was applied to further refine feature selection.

LASSO regression was performed with the glmnet (v4.1-8) package and 10-fold cross-validation to remove redundant variables and reduce overfitting [77]. Random forest analysis was performed using the randomForest (v4.7-1.2) package, and candidate genes were ranked by importance (IncNodePurity > 0.5). SVM-RFE was performed with the e1071 (v1.7-16) and caret (v7.0-1) packages to select the gene subset with optimal accuracy [78].

Core hub genes were defined as those identified by all three algorithms, with overlaps shown in a Venn diagram.

### 4.4. Construction of the PHF23-Related Prognostic Signature

Variance inflation factors (VIFs) were calculated for the hub genes, and those with VIF < 5 were retained. A multivariate Cox proportional hazards model was then established using bidirectional stepwise selection based on the Akaike information criterion (AIC).

Patient-specific risk scores were calculated using the following equation:

$$Risk\;Score=\sum_{i=1}^{n}\left(Coefficient_{i}\times Expression_{i}\right)$$

where $Coefficient_{i}$ represents the regression coefficient of gene i from the multivariate Cox model, and $Expression_{i}$ denotes its corresponding expression level.

Based on the median risk score, patients were divided into high- and low-risk cohorts. Kaplan–Meier curves and time-dependent ROC analyses were used to assess the predictive performance of the model, with analyses conducted using the survival (v3.8-3), survminer (v0.5.0), and survivalROC (v1.0.3.1) packages in R (v4.3.3).

### 4.5. Independent Prognostic Analysis and Nomogram Construction

Cox regression analyses, both univariate and multivariate, were performed to assess the risk score’s prognostic value while controlling for clinical factors. The rms (v6.7-1) R package was employed to construct a nomogram for 1-, 3-, and 5-year survival prediction. Calibration curves and DCA were used to evaluate model accuracy and clinical utility [42], and the C-index was calculated to measure predictive performance.

### 4.6. Molecular Subtype Based on PHF23-RPS Identification and Validation

Molecular subgroups were defined via unsupervised clustering using ConsensusClusterPlus (v1.66.0). The PHF23-RPS matrix was Z-score transformed and clustered by k-means (Euclidean distance) with 1000 resampling iterations (80% sampling). The optimal *k* (*k* = 2) was selected based on consensus matrices and CDF plots. PCA was used to confirm transcriptomic separation. Kaplan–Meier analysis with the log-rank test compared survival, and associations with clinicopathological features were evaluated using the Wilcoxon rank-sum test and visualized by heatmaps.

### 4.7. Immune Landscape and Microenvironment Characterization

Three computational frameworks were used to characterize the TME. ESTIMATE (v1.0.13) generated Immune, Stromal, and ESTIMATE scores to assess non-tumor cell infiltration [79]. CIBERSORT (LM22, 1000 permutations, v0.1.0) estimated proportions of 22 immune cell types [80]. GSVA (v1.50.5) evaluated enrichment of 50 hallmark gene sets from MSigDB [81]. TMB data were retrieved from cBioPortal [82]. Differences in TMB and immune checkpoint expression between subtypes were tested with the Wilcoxon rank-sum test, and Kaplan–Meier analysis was applied for combined stratification by subtype and TMB.

### 4.8. Pharmacosensitivity Screening and Molecular Docking

Drug sensitivity was predicted using the oncoPredict R package (v1.2) [83]. IC50 values for a range of compounds were estimated with the GDSC2 dataset [84] as the training set. The calcPhenotype function was applied to infer IC50 values for glioma patients, with batch effects corrected using the Empirical Bayes method. Candidate therapeutic agents were prioritized based on a significant negative correlation between predicted IC50 and the PHF23-RPS risk score.

Molecular docking was carried out using the CB-Dock2 server (http://183.56.231.194:8001/cb-dock2/index.php, accessed on 8 March 2026), an automated blind docking tool [85]. The 3D crystal structure of HAVCR2 (TIM-3; PDB ID: 7M3Z) was used as the receptor, and Entospletinib (PubChem CID: 59473233) was used as the ligand. CB-Dock2 identified potential binding pockets using the CurvCa algorithm and performed docking with AutoDock Vina (v1.1.2) [86]. The binding pose with the lowest Vina score was selected, and interactions, including hydrogen bonds and hydrophobic contacts with key residues, were analyzed and visualized via the CB-Dock2 platform.

### 4.9. Molecular Dynamics Simulation

MD simulations of the HAVCR2-Entospletinib complex were performed using GROMACS 2022 (v2022; Science for Life Laboratory, Stockholm, Sweden). The protein and ligand were parameterized using the AMBER14SB and GAFF2 force fields, respectively, with ligand partial charges assigned via the RESP method using sobtop (v1.0; Beijing Ke-yin Scientific and Technology Research Centre, Beijing, China). The complex was solvated in a 1.0 nm cubic box with the TIP3P water model and neutralized using 0.15 M NaCl. The system underwent a three-stage energy minimization (solute-constrained, ion-constrained, and fully unconstrained), utilizing 3000 steepest descent steps followed by 2000 conjugate gradient steps. Production MD was performed under the isothermal-isobaric (NPT) ensemble for 100 ns (2 fs time step). Temperature and pressure were maintained at 310 K and 1.0 bar using the Nosé-Hoover thermostat and Parrinello-Rahman barostat, respectively. Long-range electrostatics were treated using the particle mesh Ewald (PME) method (1.0 nm cutoff), and hydrogen-involved bonds were constrained by the LINCS algorithm.

### 4.10. Trajectory Analysis and MM/PBSA Calculation

Post-simulation analyses, including RMSD, RMSF, Rg, SASA, and intermolecular hydrogen bonds, were performed using standard GROMACS toolkits. The FEL was mapped using the gmx sham tool based on RMSD and Rg coordinates. Finally, the total binding free energy and per-residue energy decomposition were calculated using the MM/PBSA method via the gmx_mmpbsa (v1.6.0; Valdés-Tresanco et al.) script.

### 4.11. Cell Culture and Stable PHF23 Knockdown

Human glioma cell lines U87MG and U251 were obtained from Servicebio (Wuhan, China). The identities of the cell lines were authenticated by short tandem repeat (STR) profiling. Cells were cultured in DMEM (Gibco, Grand Island, NY, USA, Cat# 11965092) supplemented with 10% fetal bovine serum (Gibco, Grand Island, NY, USA, Cat# 10099141C) and 1% penicillin–streptomycin (Procell System^®^, Wuhan, China, Cat# PB180120) at 37 °C in a humidified incubator with 5% CO_2_.

Stable knockdown of PHF23 was achieved using a lentiviral vector carrying a specific shRNA sequence (5′-GACCGAAAGAACCGAAAGUTT-3′) under a U6 promoter, with ZsGreen as a fluorescent marker and puromycin resistance for selection (Hanbio Biotechnology, Shanghai, China). This sequence was selected based on its previously validated high knockdown efficiency and specificity in Wang et al. (2014), where it was shown to effectively modulate PHF23 levels [87]. Lentiviral particles were produced at a titer of 1 × 10^8^ TU/mL. Cells were infected with shPHF23 or non-targeting shNC lentivirus at an appropriate MOI.

After 72 h of infection, cells were selected with 2 μg/mL puromycin (Beyotime, Shanghai, China, Cat# ST551-10mg) for 14 days, and infection efficiency was tracked via ZsGreen fluorescence. Knockdown of PHF23 was confirmed by Western blot and qRT-PCR.

### 4.12. EdU Proliferation Assay

Proliferation of U87MG and U251 cells, with or without PHF23 knockdown, was evaluated using an EdU Cell Proliferation Kit (Sangon Biotech, Shanghai, China, Cat# E607204). Cells were seeded into 96-well plates at a density of 5000 cells per well and allowed to adhere overnight. After 24 h of incubation, cells were incubated with EdU reagent for 2 h, fixed with 4% paraformaldehyde, and stained according to the manufacturer’s instructions. The proliferation rate was determined as the percentage of EdU-positive cells relative to total nuclei.

### 4.13. Colony Formation Assay

For long-term proliferation assessment, U87MG and U251 cells transfected with shPHF23 or shNC were seeded into 6-well plates at a density of 500 cells per well and cultured for 14 days. During the incubation, the culture medium was refreshed every 3 days. Colonies were then fixed with methanol, stained with 0.1% crystal violet, rinsed with PBS, imaged, and quantified using ImageJ software (v1.53t; NIH, Bethesda, MD, USA).

### 4.14. Apoptosis Analysis

Apoptosis in U87MG and U251 cells transfected with shPHF23 or shNC was measured using an Annexin V-APC/7-AAD kit (MultiSciences, Hangzhou, China, Cat# AP105) by flow cytometry. Cells (including those in the culture supernatant) were harvested with EDTA-free trypsin (Beyotime, Shanghai, China, Cat# C0205) and adjusted to a density of 5 × 10^5^ cells per sample. The cells were then washed with pre-cooled PBS and resuspended in 500 μL of 1 × binding buffer. Subsequently, the cells were stained in the dark for 15 min at room temperature. Total apoptosis, including early and late stages, was analyzed with CytExpert software (v2.4.0.28).

### 4.15. Wound Healing Assay

Horizontal migration of U87MG and U251 cells transfected with shPHF23 or shNC was assessed using a wound healing assay. Cells were seeded into 6-well plates at a density of 6 × 10^5^ cells per well and grown to confluence, after which a linear scratch was made with a sterile 200 μL pipette tip. After washing twice with PBS to remove debris, cells were cultured in serum-free medium. Images were captured at 0 and 24 h to quantify wound closure.

### 4.16. Transwell Migration and Invasion Assays

Transwell assays (24-well, Corning, Corning, NY, USA, Cat# 3422) were used to evaluate cell migration and invasion. For migration, 2.0 × 10^4^ cells in 200 μL serum-free medium were added to the upper chamber, with 800 μL of medium containing 10% FBS in the lower chamber as a chemoattractant. For invasion assays, the upper chamber membranes were coated with Matrigel (Corning, Corning, NY, USA, Cat# 356230). After 24 h, cells on the lower surface were fixed with methanol, stained with 0.1% crystal violet, and counted.

### 4.17. mRNA Extraction and qRT-PCR

Total RNA was extracted from approximately 2 × 10^6^ shPHF23- or shNC-transfected glioma cells using TRIzol reagent (Thermo Fisher Scientific, Waltham, MA, USA, Cat# 15596026). Subsequently, cDNA was synthesized and mRNA levels were quantified with a Takara qRT-PCR Detection Kit (Takara, Tokyo, Japan, Cat# RR047A/RR820A) following the manufacturer’s protocol. The specific primer sequences (Sango Biotech, Shanghai, China) were as follows: PHF23 forward 5′-TGGACGAGGACATCATGGTA-3′ and reverse 5′-ACAGAGTGGGGAGGAAGGAT-3′; GAPDH forward 5′-GCCATCACAGCAACACAGAA-3′ and reverse 5′-GCCATACCAGTAAGCTTGCC-3′. Amplification was performed on a CFX96 Real-Time PCR System (Bio-Rad, Hercules, CA, USA), with GAPDH serving as the internal control. Relative expression levels were calculated using the 2^−ΔΔCt^ analytical method.

### 4.18. Western Blotting Analysis

Protein expression was assessed in shPHF23- or shNC-transfected U87MG and U251 cells. Briefly, approximately 2 × 10^6^ cells per group were harvested and lysed in RIPA buffer (50 mM Tris pH 7.4, 150 mM NaCl, 1% Triton X-100, 1% sodium deoxycholate, 0.1% SDS, and various inhibitors; Beyotime, Shanghai, China, Cat#P0013B) supplemented with 1 mM EDTA and a 1% protease inhibitor cocktail (100× stock composition: 200 mM AEBSF, 30μM Aprotinin, 13 mM Bestatin, 1.4 mM E64, 1 mM Leupeptin in DMSO; Beyotime, Shanghai, China, Cat#P1005), followed by sonication. Lysates were cleared by centrifugation at 12,000× *g* at 4 °C for 15 min. Protein concentrations in the supernatants were determined using a BCA Protein Assay Kit (Thermo Fisher Scientific, Waltham, MA, USA, Cat#A55865). Proteins were then denatured by boiling for 10 min. Equal amounts of protein were resolved by SDS-PAGE, transferred to 0.45 μm PVDF membranes (Millipore, Burlington, MA, USA), and blocked with QuickBlock™ Western blocking buffer (Beyotime, Shanghai, China, Cat#P0252) for 20 min at room temperature. The membranes were then probed with primary antibodies against PHF23 (Abcam, Cambridge, UK, Cat#ab264292, 1:2000) and GAPDH (Proteintech, Wuhan, China, Cat#10494-1-AP, 1:20,000) overnight at 4 °C. Subsequently, the membranes were incubated with Multi-rAb^®^ HRP-conjugated recombinant secondary antibodies (Proteintech, Wuhan, China, Cat#RGAM001 or RGAR001, 1:5000) for 1 h at room temperature. Protein bands were visualized using an enhanced chemiluminescence (ECL) detection system (Biosharp, Hefei, China, Cat# BL523B) and captured with an ImageQuant LAS imaging system (GE Healthcare, Chicago, IL, USA).

### 4.19. Immunohistochemistry (IHC)

Following formalin fixation and paraffin embedding, tissue sections were subjected to IHC staining to evaluate the protein expression levels. For PHF23, protein expression was quantified using the H-score system. The proliferative index was assessed via Ki-67 staining, which was recorded simply as the percentage of positive nuclei relative to the total number of tumor cells. anti-PHF23 (Abcam, Cambridge, UK, Cat#ab264292, 1:1000) and anti-Ki-67 (Proteintech, Wuhan, China, Cat#27309-1-AP, 1:4000).

### 4.20. Orthotopic Xenograft Mouse Model and In Vivo Imaging

Male BALB/c nude mice (8 weeks old, 20–25 g) were obtained from the Lanzhou Veterinary Research Institute, Chinese Academy of Agricultural Sciences. A total of 12 mice were used and randomly assigned to two groups (*n* = 6 per group) according to a random number table: shNC and shPHF23. The sample size was determined based on previous experience with this orthotopic model to ensure sufficient statistical power while minimizing animal use. Mice were acclimatized to the laboratory environment for 7 days before surgery. Animals were housed in a temperature-controlled environment (22 ± 2 °C) with a 12 h light/dark cycle and provided with free access to food and water. For orthotopic tumor establishment, 1 × 10^6^ U251 cells with PHF23 knockdown or control shRNA were stereotaxically injected into the right striatum. To minimize confounders, the treatment order was randomized and mice were housed in mixed cages. During surgery, mice were anesthetized with 3% pentobarbital sodium (50 mg/kg, intraperitoneal injection, Sigma-Aldrich, St. Louis, MO, USA, Cat# P3761). Following the procedure, mice were monitored every other day for body weight and general health. Humane endpoints were defined as >20% weight loss or severe distress, at which point mice were euthanized via anesthesia overdose. On day 14 post-implantation, intracranial tumor growth was evaluated using a 9.4 T small-animal MRI system (United Imaging Life Science Instrument, Wuhan, China). To comply with high-magnetic field safety protocols, mice were continuously anesthetized using isoflurane (1.5–2%, RWD Life Science, Shenzhen, China, Cat# R510-22) via an MRI-compatible face mask. Animals were immobilized on a specialized non-magnetic bed with a head fixation system to minimize motion artifacts. Throughout the scanning procedure, physiological vital signs were continuously monitored using an MRI-compatible system, and body temperature was maintained using a heating pad. MRI image acquisition and analysis were performed by researchers blinded to the group allocation. All animals survived to the endpoint, and no animals or data points were excluded from the analysis.

### 4.21. Tumor Volume and Histopathological Analysis

Intracranial tumor volume on day 14 was defined as the primary outcome measure. Secondary outcomes included body weight, tumor morphology (H&E), and IHC analysis of PHF23 and proliferative markers (Ki-67). Intracranial tumor dimensions were measured from MRI cross-sectional images, and tumor volume was calculated using the formula: volume (mm^3^) = (width^2^ × length)/2. At the end of the observation period, in strict accordance with the approved animal ethics protocol, all mice were euthanized via an intraperitoneal injection of anesthesia overdose (3% pentobarbital sodium, Sigma-Aldrich, St. Louis, MO, USA, Cat# P3761). Death was confirmed by the cessation of heartbeat and respiration. Brains containing the xenografts were carefully excised. Tissues were immediately fixed in 4% paraformaldehyde, embedded in paraffin, and sectioned for histological analysis. H&E staining was performed to assess tumor morphology and IHC was performed to evaluate proliferative activity and PHF23 knockdown. A digital microscope was used to image all slides for subsequent quantitative analysis.

### 4.22. Morphometric Analysis

ImageJ software (v1.53t; NIH, Bethesda, MD, USA) was used for quantitative morphometric analyses. For in vitro cellular assays, the percentage of EdU-positive cells (relative to total nuclei) and the absolute number of migrating/invading cells in Transwell assays were quantified across five randomly selected microscopic fields per well. For the wound healing assay, the cell-free gap area was measured, while for the colony formation assay, the total number of colonies per well was quantified. For clinical and xenograft IHC, five non-overlapping microscopic fields were randomly captured per section. The expression levels of PHF23 and the Ki-67 proliferation index were semi-quantitatively evaluated by assessing the staining intensity and the proportion of positively stained nuclei. All evaluations were performed by two independent, blinded investigators.

### 4.23. Statistical Analysis

R (v4.3.3) and GraphPad Prism (v10.1.2) were used for data analysis and visualization. All in vitro experiments were independently repeated three times. For bioinformatic analysis, gene expression data were log2-transformed and normalized, and results are presented as box plots showing the median and interquartile range (IQR). Quantitative results for in vitro and in vivo experiments are presented as mean ± standard deviation (SD). Data normality was assessed using the Shapiro–Wilk test, and homogeneity of variance was evaluated using Levene’s test. Statistical comparisons between two groups were conducted using Student’s *t*-test or the Wilcoxon test based on the data distribution. For multiple-group comparisons, one-way analysis of variance (ANOVA) followed by Tukey’s post hoc test, or the Kruskal–Wallis test followed by pairwise Wilcoxon tests with Benjamini–Hochberg correction, was applied where appropriate. To analyze data involving two independent variables, such as time-course measurements of body weight, a two-way ANOVA was utilized. Survival curves were estimated using the Kaplan–Meier method and compared via the log-rank test. A *p*-value < 0.05 was considered significant.

## 5. Conclusions

In conclusion, this study identified PHF23 as a crucial oncogenic driver that contributes to an “inflamed yet immunosuppressive” microenvironment in glioma. The PHF23-RPS provides a valuable tool for risk stratification. Furthermore, our integrated computational and structural analyses identify Entospletinib as a promising pharmacological agent that may attenuate immune evasion and improve therapeutic outcomes for patients within the high-risk subgroup.

## Data Availability

The datasets presented in this study can be found in online repositories. The names of the repositories and accession numbers can be found in the article/Supplementary Materials. Other data, including animal experimental results, are available on reasonable request from the corresponding author.

## Supplementary Materials

The following supporting information can be downloaded at https://doi.org/10.6084/m9.figshare.31625485 (accessed on 8 March 2026).

## Abbreviations

The following abbreviations are used in this manuscript:

CDF	Cumulative distribution function
EMT	Epithelial–Mesenchymal Transition
GBM	Glioblastoma multiforme
GSVA	Gene Set Variation Analysis
ICB	Immune checkpoint blockade
OS	Overall survival
PHF23	PHD finger protein 23
PRS	Primary or recurrent status
TAMs	Tumor-associated macrophages
TME	Tumor microenvironment
